# Gender differences in prevalence and associated factors of metabolic syndrome in first-treatment and drug-naïve schizophrenia patients

**DOI:** 10.1186/s12991-023-00455-0

**Published:** 2023-06-28

**Authors:** Kuan Zeng, Shuo Wang, Lin Zhang, Yanting Zhang, Jun Ma

**Affiliations:** 1 Department of Psychiatry, Wuhan Mental Health Center, No. 89, Gongnongbing Road, Wuhan, Hubei China; 2 Wuhan Hospital for Psychotherapy, Wuhan, China; 3 Department of Psychiatry, Renmin Hospital, Wuhan University, Wuhan, China; 4 Department of Psychiatry, Suzhou Guangji Hospital, No. 11, Guangqian Road, Suzhou, Jiangsu China

**Keywords:** Gender differences, Metabolic syndrome, Schizophrenia, First-treatment, Drug-naïve

## Abstract

**Background:**

Metabolic syndromes (MetS) are clinical syndromes involving multiple pathological states with distinct gender-specific clinical patterns. As a serious disorder associated with psychiatric conditions, the prevalence of MetS is significantly higher in the population with schizophrenia (Sch). The aim of this paper is to report gender differences in the prevalence, associated factors and severity-related factors of MetS in first-treatment and drug-naïve (FTDN) patients with Sch.

**Methods:**

A total of 668 patients with FTDN Sch were included in this study. We collected socio-demographic and general clinical information on the target population, measured and evaluated common metabolic parameters and routine biochemical indicators, and assessed the severity of psychiatric symptoms using Positive and Negative Symptom Scale (PANSS).

**Results:**

In the target group, the prevalence of MetS was significantly higher in women (13.44%, 57/424) than in men (6.56%, 16/244). In the males, waist circumference (WC), fasting blood glucose (FBG), diastolic blood pressure (DBP), and triglycerides (TG) were risk factors for MetS, while systolic blood pressure (SBP), TG, total cholesterol (TC), low-density lipoprotein cholesterol (LDL-C), and platelet (PLT) were risk factors for the females. More importantly, for the females, we found that age, LDL-C, PANSS scores and blood creatinine (CRE) were risk factors for higher MetS scores, while onset age and hemoglobin (HGB) were protective factors.

**Conclusion:**

There are significant gender differences in the prevalence of MetS and its factors among patients with FTDN Sch. The prevalence of MetS is higher and the factors that influence MetS are more numerous and extensive in females. The mechanisms of this difference need further research and clinical intervention strategies should be formulated with gender differences.

## Introduction

Metabolic syndrome (MetS) is defined as a pathological condition with multiple components including insulin resistance, atherosclerotic dyslipidemia, central obesity and hypertension, which is significantly associated with an increased risk of developing diabetes and cardiovascular disease (CVD) [1]. With the development of global health care, this disease has replaced infectious diseases as the main look hazard in the modern world [2]. According to statistics, MetS affects about a quarter of the world's population [3] and an epidemiological study show that its prevalence is increasing every year [4, 5]. According to national stream surveys in China, the prevalence of MetS has gradually increased from 13.7% in 2000–2001 [6] to 31.1% in 2015–2017 [7]. Another important characteristic of MetS is the significant gender difference, with women having a significantly higher risk of prevalence compared to men [6–9]. Therefore, we believe that appropriate health management measures for metabolic syndrome in men and women, equally and differently, are important medical topics, especially in patients with schizophrenia (Sch), a population with a high prevalence of MetS that seems to be more critical [10].

Sch is a serious and highly disabling psychiatric disorder [11] that affects approximately 1% of the world's population [12] and reduces life expectancy by 15–20 years [13], with comorbid MetS being a significant direct or indirect contributor to this reduction [14–17]. Atypical antipsychotics (APs) are the primary and most commonly used treatment for Sch, but unacceptably, these drugs have complex mechanisms of adverse effects leading to metabolic disorders [18, 19], and the incidence of MetS in patients taking them can be as high as 22.4–63%, three times that of the general population [20]. However, it does not seem reasonable to attribute the high prevalence of MetS in patients with Sch all to APs exposure, as there are indications that metabolic disturbances in schizophrenic patients predate being prescribed antipsychotics [21]. Numerous studies have shown that even in a drug-naïve state, patients with Sch also experience significantly higher levels of insulin resistance [22, 23], high body mass index and obesity rates [24], and higher rates of impaired glucose tolerance [25] than the healthy population. It seems that we can find that Sch itself is a risk factor for MetS, or that the disease has a predisposition to MetS. Therefore, it is crucial to conduct research on the MetS for the population diagnosed with Sch.

The research on gender differences in Sch is equally compelling. Several studies have found negative symptoms more often in male patients [26], while affective symptoms are more prominent in female patients [27]. In terms of disease course, women appear to have higher remission rates, lower relapse rates [28] and a better prognosis [29] than men. Admittedly, gender differences in the metabolic disorders of Sch are of equal concern to psychiatrists. For example, male patients with a first-episode Sch are more likely than females to develop insulin resistance and abnormal lipid levels [30]. The prevalence of diabetes in patients with Sch on long-term antipsychotic medication is significantly higher in women (27%) than in men (17%) [31]. In addition, only body mass index in female patients is associated with brain-derived neurotrophic factor (BDNF) [32]. However, most of the currently known studies focus on selected indicators of MetS components and lack exploration of MetS as a whole [33, 34], or have small sample sizes [35–37], or do not explore in greater depth the factors associated with MetS severity [34, 36].

In our study, building on previous studies of gender differences in both Sch and MetS, we will report gender differences in the prevalence and influencing factors of MetS in a larger sample of patients with first-treatment and drug-naïve (FTDN) Sch and dig deeper into gender differences in factors associated with MetS severity, with a view to informing clinical interventions for MetS in this subgroup of the population across gender.

## Materials and methods

### Subjects

From February 2017 to June 2022, a total of 668 patients with FTDN Sch were enrolled at the Wuhan Mental Health Center. The mean age of the patients was 29.58 ± 7.18 years, and 63.47% of the patients were female.

Inclusion criteria: The patients included in our study met the 10th revision of the International Classification of Diseases (ICD-10) schizophrenia diagnostic criteria. They were aged between 18 and 49 years, with no gender specification. The Positive and Negative Symptom Scales (PANSS) score, used to assess the degree of psychopathology, was at least 60. Prior to this assessment, there were no records of antipsychotic exposure, and benzodiazepines were not prohibited for use. Additionally, our investigation did not exclude patients with comorbid, unmedicated metabolic system illnesses such as hypertension, hyperlipidemia, diabetes mellitus, and obesity.

#### Exclusion criteria

All patients under the age of 18 and patients with other mental illnesses, such as bipolar disorder, depression, intellectual developmental disorders, substance abuse or dependency, were not included. Also, we disqualified those with coexisting, severe somatic illnesses, autoimmune disorders, surgeries of any kind within the last 6 months. Any patient with a previously established metabolic disorder and on therapeutic medication (e.g., antihypertensives, hypoglycemic drugs, lipid-lowering drugs, etc.) will also be excluded.

#### Withdrawal criteria

Patients who cannot be definitively diagnosed within a short period of time will be followed up for a further 14 days. If the patient is still unable to be diagnosed, then they will be withdrawn from the study.

The study was reviewed and approved by the Ethics Committee of Wuhan Mental Health Center, and all participants signed a written informed consent form.

### Research design

This cross-sectional study intended to compare gender differences in MetS prevalence and to analyze the associated factors affecting MetS and its severity among patients with FTDN Sch.

A self-made spreadsheet was used to collate the socio-demographic and general clinical information of the patients included. On the second day of the patient's admission, we extracted the patient's recurring blood, biochemical signs, thyroid function, and many others, from the digital medical file gadget. These included the patient's red blood cell (RBC), hemoglobin (HGB), white blood cell (WBC), platelet (PLT), fasting blood glucose (FBG), triglycerides (TG), high-density lipoprotein cholesterol (HDL-C), total cholesterol (TC), low-density lipoprotein cholesterol (LDL-C), blood urea nitrogen (BUN), blood creatinine (CRE), blood uric acid (UA), thyroid stimulating hormone (TSH), free triiodothyronine (FT_3_), free tetraiodothyronine (FT_4_), systolic blood pressure (SBP), diastolic blood pressure (DBP).

Diagnostic criteria of metabolic syndrome: the diagnostic criteria for metabolic syndrome in China require that at least three of the following five indicators be met [38]: 1. abdominal obesity: waist circumference (WC) ≥ 90 cm in men and ≥ 85 cm in women. 2. Hyperglycemia: FBG ≥ 6.1 mmol/L and/or those who have been diagnosed and treated for diabetes mellitus. 3. Hypertension: SBP ≥ 130/85 mmHg or DBP ≥ 85 mmHg or confirmed and treated hypertension. 4. TG ≥ 1.70 mmol/L. 5. HDL-C < 1.04 mmol/L.

Assessment of psychopathology: four uniformly trained attending psychiatrists used the PANSS and Clinical Global Impression Scale—Severity of Illness (CGI-SI) to assess the severity of psychiatric symptoms on the day of patient admission. We also divided PANSS into five factors (including positive factor, negative factor, excitement factor, anxiety/depression factor, cognitive factor) for inclusion in the statistical analysis during the actual operation, as reported by *Jong-Hoon Kim* et al. [39].

Scoring rules for MetS: based on previous studies, we have scored the severity of the MetS in clinical subgroups of the MetS [40, 41]. According to the scoring rules, we first calculated the reciprocal of HDL-C and mean average pressure (MAP). MAP was calculated as MAP = $1/3\times \mathrm{SBP}+2/3\times \mathrm{DBP}$. Then we normalized the new five parameters of the metabolic syndrome obtained (WC, TG, reciprocal of HDL-C, FBG and MAP, respectively). In the third step, the five normalized components were subjected to a principal component analysis with varimax rotation to drive PCs (eigenvalue 1.0) that accounted for a significant portion of the observed variation. In the present study, PC1 and PC2 explained 37.75% and 21.94% of the variance, respectively [loadings PC1 (PC2): WC 0.53 (-0.56), TG 0.66 (− 0.05), HDL 0.82 (0.14), MAP 0.30(0.87) and FBG − 0.64 (0.08)]. The weight of the PC score was determined by the relative weights of PC1 and PC2 in the explained variance. The individual weighted PC scores were then added up to create the MetS score.

### Data analysis

The categorical variables are stated in terms of counts, while the data acquired for the normally distributed continuous measures are reported in terms of mean and standard deviation. T-tests on independent samples were employed to compare continuous variable from various groups. Chi-squared tests were used to compare rates. To compare gender differences in comorbid abnormal glucose metabolism and clinical parameters, we used 2 × 2 ANOVAs, taking into account gender (2 levels: male and female) and diagnosis (2 levels: with MetS and without MetS) and the main effects of gender and subclinical groups, as well as the interaction between gender × diagnosis groups, were tested. Further, separate ANOVAs were performed in the male and female groups to compare the differences in clinical parameters between the subclinical groups that did and did not have MetS. We also calculated Pearson correlation coefficients to assess the association between MetS and the other variables (exclusion of MetS components). And then, binary logistic regression analysis was used to explore the correlations of MetS in the included samples both the males and females. Finally, multivariate linear regression models were constructed to analyze the factors associated with MetS scores in the males and females separately. All *p* values were 2-tailed, and the significance level was < 0.05. Statistical analyses were performed using SPSS 27 (SPSS, Inc., Chicago, IL).

## Results

### Demographic and general clinical data of enrolled patients

The demographic data and general clinical data of the included patients, as well as the gender differences between these data, are shown in Table 1. Compared to male, the WC was significantly higher (t =− 2.05, *p* = 0.041), and the CGI-SI was lower (t = 2.21, *p* = 0.035) in female patients.

**Table 1:** Gender differences in demographic and general clinical data

Index	Total patients (n = 668)	Male (n = 244)	Female (n = 424)	$t{/\chi }^{2}$	*p*-value
Age—years	29.58 ± 7.18	29.23 ± 6.87	29.77 ± 7.35	− 0.94	0.348
Course of disease—years	5.14 ± 4.11	5.26 ± 4.6	5.07 ± 4.78	0.49	0.623
Onset age—years	24.44 ± 6.40	23.98 ± 6.32	24.7 ± 6.43	− 1.42	0.157
Marital status—(n, %)				0.64	0.424
Spousal	312, 46.71%	109, 44.67%	203, 47.88%		
Others	356, 53.29%	135, 55.33%	221, 52.12%		
Educational background—(n, %)				3.56	0.059
Junior school and below	440, 65.87%	158, 64.75%	282, 66.51%		
High school and above	228, 34.13%	86, 35.25%	142, 33.49%		
MetS dimensions					
WC—cm	78.32 ± 9.05	77.38 ± 9.06	78.87 ± 9.01	− 2.05	0.041*
FBG—mmol/L	5.57 ± 0.49	5.55 ± 0.5	5.59 ± 0.48	− 1.00	0.319
SBP—mmHg	113.08 ± 12.36	112.64 ± 11.53	113.34 ± 12.82	− 0.71	0.477
DBP—mmHg	75.16 ± 8.71	75.08 ± 7.95	75.2 ± 9.12	− 0.18	0.861
TG—mmol/L	1.09 ± 0.55	1.08 ± 0.55	1.1 ± 0.56	− 0.36	0.720
HDL-C—mmol/L	1.18 ± 0.23	1.18 ± 0.25	1.19 ± 0.22	− 0.40	0.689
TC—mmol/L	3.85 ± 0.72	3.84 ± 0.7	3.85 ± 0.72	− 0.16	0.875
LDL-C—mmol/L	2.18 ± 0.58	2.19 ± 0.58	2.18 ± 0.59	0.28	0.778
RBC—10^12^/L	4.55 ± 0.47	4.52 ± 0.51	4.56 ± 0.45	− 1.05	0.293
HGB—g/L	135.92 ± 17.32	135.44 ± 18.28	136.2 ± 16.76	− 0.55	0.584
WBC—10^9^/L	6.91 ± 2.00	7.07 ± 2.15	6.82 ± 1.91	1.52	0.130
PLT—10^9^/L	242.33 ± 57.46	244.59 ± 60.17	241.03 ± 55.87	0.77	0.440
BUN—mmol/L	4.49 ± 1.77	4.59 ± 1.76	4.43 ± 1.77	1.18	0.239
CRE—mmol/L	57.92 ± 12.56	58.07 ± 12.41	57.83 ± 12.66	0.24	0.814
UA—mmol/L	353.19 ± 123.88	418.34 ± 128.97	410.23 ± 120.91	0.81	0.416
TSH—uIU/mL	1.70 ± 0.72	1.72 ± 0.71	1.68 ± 0.72	0.70	0.481
FT_3_—pmol/L	4.85 ± 0.69	4.88 ± 0.68	4.83 ± 0.69	0.79	0.433
FT_4_—pmol/L	16.94 ± 3.15	17.03 ± 3.21	16.9 ± 3.13	0.51	0.609
CGI-SI	5.36 ± 0.62	5.43 ± 0.62	5.32 ± 0.62	2.12	0.035*
PANSS	88.90 ± 11.43	89.43 ± 11.52	88.6 ± 11.38	0.90	0.367
Positive factor	15.90 ± 3.51	15.82 ± 3.57	15.94 ± 3.48	− 0.42	0.678
Negative factor	28.23 ± 6.22	28.64 ± 6.39	27.99 ± 6.12	1.29	0.199
Excitement factor	12.71 ± 4.44	12.85 ± 4.4	12.63 ± 4.46	0.60	0.549
Anxiety/depression factor	15.43 ± 4.50	15.21 ± 4.71	15.55 ± 4.38	− 0.92	0.356
Cognitive factor	16.48 ± 4.41	16.68 ± 4.67	16.36 ± 4.25	0.91	0.361

*WC* waist circumference, *FBG* fasting blood glucose, *SBP* systolic blood pressure, *DBP* diastolic blood pressure, *TG* triglycerides, *HDL-C* high-density lipoprotein cholesterol, *TC* total cholesterol, *LDL-C* low-density lipoprotein cholesterol, *RBC* red blood cell, *HGB* hemoglobin, *WBC* white blood cell, *PLT* platelet, *BUN* blood urea nitrogen, *CRE* blood creatinine, *UA* blood uric acid, *TSH* thyroid stimulating hormone, *FT*_*3*_ free triiodothyronine, *FT*_*4*_ free tetraiodothyronine, *PANSS* Positive and Negative Syndrome Scale, *CGI-SI* Clinical Global Impression Scale—Severity of Illness. *p < *0.05*

### Gender differences in the prevalence, demographic and clinical features of MetS in Sch patients

The overall prevalence of MetS in our included sample was 10.93% (73/668), with 6.56% (16/244) of male patients and 13.44% (57/424) of female patients. The prevalence was significantly higher in females than in males (${\chi }^{2}$= 7.54, *p* = 0.006). The MetS-*z* score for males with MetS was (− 0.27 ± 0.89) and for females was (0.09 ± 0.78), with no gender differences in MetS-*z* scores (t = − 1.54, *p* = 0.128). As shown in Table 2, ANOVA was performed to examine the interaction between MetS and gender. There was extensive difference in various clinical parameters between different clinical subgroups, for example WC (F = 95.45, *p* < 0.001), FBG (F = 8.24, *p* = 0.004), SBP (F = 53.71, *p* < 0.001), DBP (F = 80.06, *p* < 0.001), TG (F = 74.79, *p* < 0.001), TC (F = 33.95, *p* < 0.001), LDL-C (F = 23.22, *p* < 0.001), RBC (F = 6.89, *p* = 0.009), HGB (F = 8.35, *p* = 0.004), WBC (F = 9.02, *p* = 0.003), PLT (F = 5.20, *p* = 0.023), FT_4_ (F = 19.35, *p* < 0.001), CGI-SI (F = 10.63, *p* = 0.001). Meanwhile, gender × subgroup had an effect on age (F = 7.24, *p* < 0.001), onset age (F = 5.59, *p* = 0.018) and SBP (F = 5.23, *p* = 0.023).

**Table 2:** Demographic and clinical characteristics between enrolled patients with and without MetS, grouped by gender

Parameters	MetS (*n* = 73)		Non-MetS (*n* = 595)		Gender F (*p* -value)	Diagnosis F (*p* -value)	Gender × diagnosis F (*p* -value)
	Male (*n* = 16)	Female (*n* = 57)	Male (*n* = 228)	Female (*n* = 367)			
Age—years	31.08 ± 7.72	26.59 ± 5.55	29.10 ± 6.80	30.27 ± 7.47	2.50(0.114)	0.66 (0.419)	7.24 (< .001*)
Course of disease—years	6.70 ± 5.30	5.33 ± 3.88	5.15 ± 4.55	5.03 ± 4.91	1.16 (0.283)	1.76 (0.185)	0.80(0.371)
Onset age—years	24.37 ± 5.77	21.26 ± 4.69	23.95 ± 6.36	25.24 ± 6.51	0.96 (0.328)	3.63 (0.057)	5.59 (0.018*)
MetS dimensions							
WC—cm	86.53 ± 10.3	90.64 ± 6.52	76.74 ± 8.63	77.04 ± 7.89	3.39 (0.066)	95.45 (< .001*)	2.53 (0.112)
FBG—mmol/L	5.87 ± 0.50	5.65 ± 0.57	5.53 ± 0.49	5.58 ± 0.46	1.42 (0.234)	8.24 (0.004*)	3.70 (0.055)
SBP—mmHg	120.63 ± 14.82	127.46 ± 14.91	112.07 ± 11.09	111.15 ± 10.96	3.03 (0.082)	53.71(< .001*)	5.23 (0.023*)
DBP—mmHg	84.38 ± 10.94	84.95 ± 10.89	74.43 ± 7.29	73.69 ± 7.81	0.01 (0.944)	80.06 (< .001*)	0.31 (0.580)
TG—mmol/L	1.77 ± 0.55	1.73 ± 0.60	1.04 ± 0.51	1.00 ± 0.49	0.21 (0.646)	94.79 (< .001*)	0.00 (0.997)
HDL-C—mmol/L	1.22 ± 0.19	1.15 ± 0.14	1.17 ± 0.26	1.19 ± 0.23	0.53 (0.467)	0.00 (0.950)	1.41 (0.236)
TC—mmol/L	4.38 ± 0.70	4.39 ± 0.67	3.80 ± 0.69	3.77 ± 0.7	0.02 (0.8901)	33.95 (< .001*)	0.05 (0.828)
LDL-C—mmol/L	2.57 ± 0.81	2.53 ± 0.65	2.16 ± 0.55	2.12 ± 0.56	0.19 (0.661)	23.22 (< .001*)	0.00 (0.950)
RBC—10^12^/L	4.62 ± 0.30	4.78 ± 0.32	4.51 ± 0.52	4.53 ± 0.46	1.53 (0.216)	6.89 (0.009*)	1.05 (0.306)
HGB—g/L	139.62 ± 12.57	144.96 ± 12.08	135.14 ± 18.6	134.84 ± 16.98	0.99 (0.320)	8.35 (0.004*)	1.25 (0.264)
WBC—10^9^/L	7.59 ± 2.28	7.86 ± 1.95	7.03 ± 2.14	6.66 ± 1.85	0.03 (0.866)	9.02 (0.003*)	1.19 (0.275)
PLT—10^9^/L	263.81 ± 82.21	256.58 ± 52.17	243.25 ± 58.32	238.61 ± 56.1	0.49 (0.483)	5.20 (0.023*)	0.24 (0.878)
BUN—mmol/L	4.28 ± 1.22	4.08 ± 1.13	4.61 ± 1.79	4.48 ± 1.85	0.41 (0.524)	2.02 (0.156)	0.01 (0.908)
CRE—mmol/L	55.56 ± 17.42	61.78 ± 18.09	58.25 ± 12.01	57.22 ± 11.51	1.99 (0.160)	0.26 (0.613)	3.85 (0.050)
UA—mmol/L	425.4 ± 129.32	401.12 ± 83.15	417.84 ± 129.21	411.65 ± 125.79	0.64 (0.405)	0.01 (0.936)	0.25 (0.621)
TSH—uIU/mL	1.83 ± 0.73	1.74 ± 0.70	1.71 ± 0.71	1.67 ± 0.72	0.37 (0.543)	0.75 (0.388)	0.04 (0.835)
FT_3_—pmol/L	4.84 ± 0.75	4.68 ± 0.77	4.88 ± 0.67	4.86 ± 0.68	0.80 (0.372)	1.21 (0.271)	0.46 (0.498)
FT_4_—pmol/L	14.73 ± 2.32	15.53 ± 3.30	17.19 ± 3.20	17.11 ± 3.05	0.63 (0.429)	19.35 (< .001*)	0.93 (0.335)
CGI-SI	5.13 ± 0.72	5.09 ± 0.43	5.45 ± 0.61	5.36 ± 0.64	0.50 (0.482)	10.63 (0.001*)	0.09 (0.770)
PANSS	90.69 ± 14.54	84.74 ± 11.6	89.34 ± 11.32	89.2 ± 11.24	3.29 (0.070)	0.86 (0.354)	2.99 (0.084)
Positive factor	16.31 ± 3.24	15.18 ± 3.17	15.79 ± 3.60	16.06 ± 3.51	0.70 (0.403)	0.12 (0.727)	1.85 (0.174)
Negative factor	29.06 ± 7.19	27.09 ± 7.14	28.61 ± 6.34	28.13 ± 5.94	1.78 (0.183)	0.10 (0.749)	0.67 (0.413)
Excitement factor	12.44 ± 5.35	11.33 ± 4.64	12.88 ± 4.33	12.84 ± 4.41	0.77 (0.381)	2.21 (0.138)	0.66 (0.416)
Anxiety/depression factor	14.31 ± 4.29	14.30 ± 3.97	15.28 ± 4.74	15.74 ± 4.41	0.12 (0.734)	3.30 (0.070)	0.13 (0.718)
Cognitive factor	17.94 ± 5.78	16.53 ± 3.86	16.6 ± 4.58	16.34 ± 4.31	1.65 (0.199)	1.39 (0.240)	0.78 (0.377)

*WC* waist circumference, *FBG* fasting blood glucose, *SBP* systolic blood pressure, *DBP* diastolic blood pressure, *TG* triglycerides, *HDL-C* high-density lipoprotein cholesterol, *TC* total cholesterol, *LDL-C* low-density lipoprotein cholesterol, *RBC* red blood cell, *HGB* hemoglobin, *WBC* white blood cell, *PLT* platelet, *BUN* blood urea nitrogen, *CRE* blood creatinine, *UA* blood uric acid, *TSH* thyroid stimulating hormone, *FT*_*3*_ free triiodothyronine, *FT*_*4*_ free tetraiodothyronine, *PANSS* Positive and Negative Syndrome Scale, *CGI-SI* Clinical Global Impression Scale—Severity of Illness. *p < *0.05*

### The related factors of MetS in male Sch patients

In the male patients, TC (r = 0.20, *p* = 0.001), LDL-C (r = 0.17, *p* = 0.017) were positively correlated with MetS, but FT_4_ (*r* = − 0.19, *p* = 0.003), CGI-SI (*r* = − 0.13, *p* = 0.044) were negatively correlated with MetS (Table 3). Further, we used MetS (marked as 0 = non-MetS, 1 = MetS) as the dependent variable, take all clinical parameters above significantly related to MetS as independent variables, and constructed a binary logistic regression model to analyze the influencing factors of MetS. It was found that TC (OR = 9.41, 95%CI 1.381–48.87, *p* = 0.008) was risk factors for MetS, while, FT_4_ (OR = 0.72, 95%CI 0.57–0.90, *p* = 0.004) and CGI-SI (OR = 0.32, 95%CI 0.12–0.85, *p* = 0.023) were predictive factor (Table 4). Finally, multiple linear regression models (Input) constructed with MetS scores as the dependent variable and clinical variables associated with MetS as the independent variables, no variables associated with MetS scores were found (Table 5).

**Table 3:** Correlation between MetS and demographic and clinical variable in male and female patients

Characteristic	Male (*n* = 244)	Female (*n* = 424)
	r(*p*)	r(*p*)
Age—years	0.07(0.267)	− 0.17(< .001*)
Course of disease—years	0.08(0.195)	0.02(0.659)
Onset age—years	0.02(0.794)	− 0.21(< .001*)
Marital status (spousal *vs.* others)	0.03(0.659)	− 0.13(0.008*)
High school and above (yes *vs.* no)	0.01(0.846)	− 0.05(0.353)
TC—mmol/L	0.20(0.001*)	0.29(< .001*)
LDL-C—mmol/L	0.17(0.007*)	0.24(< .001*)
RBC—10^12^/L	0.05(0.403)	0.19(< .001*)
HGB—g/L	0.06(0.344)	0.21(< .001*)
WBC—10^9^/L	0.06(0.317)	0.21(< .001*)
PLT—10^9^/L	0.09(0.187)	0.11(0.024*)
BUN—mmol/L	− 0.05(0.455)	− 0.08(0.113)
CRE—mmol/L	− 0.05(0.403)	0.12(0.011*)
UA—mmol/L	0.02(0.820)	− 0.03(0.542)
TSH—uIU/mL	0.04(0.538)	0.03(0.498)
FT_3_—pmol/L	− 0.02(0.807)	− 0.09(0.068)
FT_4_—pmol/L	− 0.19(0.003*)	− 0.17(< .001*)
CGI-SI	− 0.13(0.044*)	− 0.15(0.002*)
PANSS	0.03(0.653)	− 0.13(0.006*)
Positive factor	0.04(0.572)	− 0.09(0.074)
Negative factor	0.02(0.783)	− 0.06(0.230)
Excitement factor	− 0.03(0.700)	− 0.12(0.018*)
Anxiety/depression factor	− 0.05(0.429)	− 0.11(0.020*)
Cognitive factor	0.07(0.267)	0.02(0.753)

*TC* total cholesterol, *LDL-C* low-density lipoprotein cholesterol, *RBC* red blood cell, *HGB* hemoglobin, *WBC* white blood cell, *PLT* platelet, *BUN* blood urea nitrogen, *CRE* blood creatinine, *UA* blood uric acid, *TSH* thyroid stimulating hormone, *FT*_*3*_ free triiodothyronine, *FT*_*4*_ free tetraiodothyronine, *PANSS* Positive and Negative Syndrome Scale, *CGI-SI* Clinical Global Impression Scale—Severity of Illness. *p < *0.05*

**Table 4:** Binary logistic regression analyses of determinants of MetS in male and female patients

	Coefficients	Std. error	Wald	*p* value	95% CI for EXP (B)		
	B				Exp(B)	Lower	Upper
Male							
TC—mmol/L	2.24	0.84	7.12	0.008*	9.41	1.81	48.87
LDL-C—mmHg	− 1.10	0.85	1.65	0.199	0.33	0.06	1.78
FT_4_—pmol/L	− 0.34	0.12	8.12	0.004*	0.72	0.57	0.90
CGI-SI	− 1.13	0.50	5.20	0.023*	0.32	0.12	0.85
Female							
Age—years	− 0.16	0.04	20.63	< .001*	0.85	0.79	0.91
TC—mmol/L	1.70	0.29	34.55	< .001*	5.47	3.10	9.64
RBC—10^12^/L	1.89	0.46	16.60	< .001*	6.59	2.66	16.33
WBC—10^9^/L	0.34	0.10	12.38	< .001*	1.40	1.16	1.69
FT_4_—pmol/L	− 0.16	0.06	7.06	0.008*	0.85	0.76	0.96
CGI-SI	− 1.57	0.35	20.45	< .001*	0.21	0.11	0.41

*TC* total cholesterol, *LDL-C* low-density lipoprotein cholesterol, *FT*_*4*_ free tetraiodothyronine, *CGI-SI* Clinical Global Impression Scale—Severity of Illness, *TC* total cholesterol, *RBC* red blood cell, *WBC* white blood cell. *p < 0.05

**Table 5:** Correlates affecting MetS scores in male and female patients: a multiple linear regression model

	Coefficients	Std. error	*t*	*p-*value	95% CI	
	B				Lower	Upper
Male						
TC—mmol/L	0.13	0.73	0.18	0.858	− 1.48	1.74
LDL-C—mmol/L	− 0.07	0.64	− 0.11	0.915	− 1.47	1.33
FT_4_—pmol/L	− 0.11	0.13	− 0.87	0.401	− 0.39	0.17
CGI—SI	− 0.01	0.41	− 0.02	0.988	− 0.90	0.89
Female						
Age—years	0.10	0.03	3.31	0.002*	0.04	0.15
Onset age—years	− 0.07	0.03	− 2.10	0.041*	− 0.14	0.00
LDL-C—mmol/L	0.37	0.16	2.32	0.024*	0.05	0.69
HGB—g/L	− 0.05	0.01	− 3.41	0.001*	− 0.07	-0.02
WBC—10^9^/L	0.12	0.06	1.91	0.062	− 0.01	0.24
CRE—mmol/L	0.03	0.01	3.76	< .001*	0.01	0.04
PANSS	0.03	0.01	3.64	0.001*	0.02	0.05

*TC* total cholesterol, *LDL-C* low-density lipoprotein cholesterol, *FT*_*4*_ free tetraiodothyronine, *CGI-SI* Clinical Global Impression Scale—Severity of Illness, *HGB* hemoglobin, *WBC* white blood cell, *CRE* blood creatinine, *PANSS* Positive and Negative Syndrome Scale. *p < 0.05

### The related factors of MetS in female Sch patients

In the female patients, TC (*r* = 0.29, *p* < 0.001), LDL-C (*r* = 0.24, *p* < 0.001), RBC (*r* = 0.19, *p* < 0.001), HGB (*r* = 0.21, *p* < 0.001), WBC (*r* = 0.21, *p* < 0.001), PLT (*r* = 0.11, *p* = 0.024), CRE (*r* = 0.12, *p* = 0.011) were positively correlated with MetS, but age (*r* = − 0.17, *p* < 0.001), onset age (*r* = − 0.21, *p* < 0.001), FT_4_ (*r* = 0.43, *p* < 0.001), Marital status (spousal vs. others) (*r* = -0.13, *p* = 0.008), CGI-SI (*r* = − 0.15, *p* = 0.002), PANSS (*r* = − 0.13, *p* = 0.006), excitement factor (*r* = − 0.12, *p* = 0.018), anxiety/depression factor (*r* = − 0.11, *p* = 0.018) were negatively correlated with MetS (Table 3). Further, we used MetS as the dependent variable, take the above clinical parameters related to MetS as independent variables, and constructed a binary logistic regression model to analyze the influencing factors of MetS. It was found that TC (OR = 5.47, 95%CI 3.10–9.64,* p* < 0.001), RBC (OR = 6.59, 95%CI 2.66–16.33, *p* < 0.001), WBC (OR = 1.40, 95%CI 1.16–1.69, *p* < 0.001) were independent predictors of MetS, while, age (OR = 0.85, 95%CI 0.79–0.91, *p* < 0.001), FT_4_ (OR = 0.85, 95%CI 0.76–0.96, *p* = 0.008) and CGI-SI (OR = 0.21, 95%CI 0.11–0.41, *p* < 0.001) were predictive factors (Table 4). Finally, multiple linear regression models constructed with MetS scores as the dependent variable and clinical variables associated with MetS as the independent variables, We found that age (*B* = 0.10, *t* = 3.31, *p* = 0.002), LDL-C (*B* = 0.37, *t* = 2.32, *p* = 0.024), CRE (*B* = 0.03, *t* = 3.76, *p* < 0.001) and PANSS (*B* = 0.03, *t* = 3.64, *p* = 0.001) were risk factors for higher MetS scores, while onset age (*B* = − 0.07, *t* = − 2.10, *p* = 0.041) and HGB (*B* = − 0.05, *t* = − 3.41, *p* = 0.001) were protective factors for the higher MetS score (Table 5).

## Discussion

The present study analyzed and reported gender differences in the prevalence of MetS and the factors influencing it in patients with FTDN Sch. The main findings of our study were as follows: 1. the prevalence of MetS was significantly higher in females than in males. 2. There were gender differences in the risk factors for MetS. 3. The factors associated with the severity of MetS also differed by gender.

Several studies and meta-analyses have shown that the presence of metabolic disorders at the onset of primary schizophrenia [42–44] may be closely related to psychopathology [45] and the existence of common genetic pathways and variants in this psychiatric disorder and MetS [46, 47]. In the present study, we report a significant gender difference in the prevalence of MetS in the included sample (female *vs.* male: 13.44% *vs.* 6.56%). The small sample from Iran reported gender differences in the prevalence of MetS in patients with first-episode Sch using three different MetS diagnostic criteria (all were more higher in females) [48]. Another study with 303 Spanish FTDN Sch patients reported a significantly higher prevalence of high WC (a component of MetS) in females than in males [36]. In conclusion, the presence of sex differences in the prevalence of MetS may be an important clinical feature of FTDN Sch patients; whereas estrogen may be an important contributor in causing this difference [49, 50]. However, *Yongjie Zhou* et al. reported a higher but gender-neutral prevalence of MetS in FTDN Sch patients (female *vs.* male: 21.1% *vs.* 20.1%) [51]. This may be related to the greater severity of psychopathological symptoms [51, 52] and more stringent diagnostic criteria for MetS in the patients included in this study.

Another of our key findings is that there are gender differences in the factors influencing MetS in the target population. In terms of risk factors for MetS, in addition to TC as a common risk factor for both subgroups, females are affected by a broader range of clinical parameters, such as RBC and WBC. Similarly, for protective factors for MetS, FT_4_ and CGI-SI are common to both male and female patients, but higher age is only a protective factor for the females. Although a wider range of clinical parameters were not included as in our study, a previous study similarly reported gender differences in the contributing factors to MetS in drug naïve schizophrenia patients, and again the female subgroup had a wider range of risk factors (the contributing factors differed from those reported in ours) [51]. Some studies have reported that thyroid function [53] and negative symptoms [54] may be one of the reasons for gender differences in MetS metabolism-related parameters in patients with schizophrenia. However, unfortunately, in the present study, there were no gender differences in the three components of thyroid function (TSH, FT_3_, FT_4_) and negative symptoms in the included samples. In conclusion, the reasons for the more diverse and widespread MetS-related factors in the female subgroup of the FTDN Sch population remain to be further explored and confirmed.

The severity of MetS is a topic that is rarely addressed, especially in Sch patients, a population vulnerable to MetS. After our exploration, we found gender differences in the factors affecting MetS scores, specifically, the female subgroup presented age, LDL-C, CRE and PANSS as risk factors, while onset age, and HGB were protective factors. However, in the male subgroup, no relevant factors were found. In a large sample PREDIMED-Plus study, the investigators reported that MetS severity was associated with risk of depression [55]. Unfortunately, there were no gender differences in depression factor scores in our included sample. The National Health and Nutrition Examination Survey of the general adult population from Korea found that progression scores for MetS severity were significantly higher in women than in men [56], which would seem to provide some side support for our findings that MetS severity affects more women patients than men. Furthermore, we cannot completely deny that since the age range of our included patients was 18–49 years, sex hormones may still be an important contributor to this gender difference [57]. However, due to the inadequacy of studies involving the severity of MetS, our findings still need further validation.

Our study also has some shortcomings. Firstly, as a cross-sectional study, we were unable to determine causality and a prospective study will be set up in the future to fill this gap. Secondly, there was a gender imbalance in our included sample, meaning that the sample size of males was significantly lower than females, which may contribute to the statistical efficacy of the regression analysis for male patients.

In summary, there are significant gender differences in the prevalence of MetS and its factors among patients with FTDN Sch. The prevalence of MetS is higher and the factors that influence MetS are more numerous and extensive in females. Further prospective studies are needed in the future to determine the mechanisms and reasons for this discrepancy. In clinical practice, interventions for MetS in females need to be more targeted and specific clinical strategies than for males.

## Data Availability

The datasets used and/or analyzed during the current study are available from the corresponding author on reasonable request.

## Abbreviations

Aps: Antipsychotics
BDNF: Brain-derived neurotrophic factor
BUN: Blood urea nitrogen
CGI-SI: Clinical Global Impression Scale—Severity of Illness
CRE: Blood creatinine
CVD: Cardiovascular disease
DBP: Diastolic blood pressure
FBG: Fasting blood glucose
FTDN: First-treatment and drug-naïve
FT_3_: Free triiodothyronine
FT_4_: Free tetraiodothyronine
HDL-C: High-density lipoprotein cholesterol
HGB: Hemoglobin
ICD-10: International Classification of Diseases 10th Revision
LDL-C: Low-density lipoprotein cholesterol
MAP: Mean average pressure
MetS: Metabolic syndrome
PANSS: Positive and Negative Symptom Scales
PLT: Platelet
RBC: Red blood cell
SBP: Systolic blood pressure
Sch: Schizophrenia
TC: Total cholesterol
TG: Triglycerides
TSH: Thyroid stimulating hormone
UA: Blood uric acid
WBC: White blood cell
WC: Waist circumference
